# Constipation

**Published:** 1861-01

**Authors:** J. C. Batdorf


### CONSTIPATION.

BY J. C. BATDORF, M. D.

The following case will illustrate the course and treatment of a certain form of constipation heretofore but imperfectly understood by a large portion of the profession, for the reason, perhaps, that its occurrence is comparatively rare.

J. G., aged seven months and a half—male child—of healthy parents—weighed nine pounds at birth, appeared well in all respects until ten days old, when the bowels failing to act with their accustomed regularity, a portion of castor oil was given, producing free evacuation. From this period, the action of the bowels was irregular; from two to four days would intervene between each evacuation, if not interfered with. A physician (an empiric) was called, who prescribed a cathartic and went away. In three days was called again; another cathartic given, producing as before two or three alvine discharges; three days more having passed without defecation, he was again called; prescription as before, result the same. Thus matters continued for near three months, the child emaciating rapidly, appetite good in the main, when the gentleman in attendance was pressed for his opinion of the case which he at first termed "derangement of the liver." His diagnosis now was, "the heart disease;" prognosis, "the child will not live three months." He continued to treat the case four months longer, giving, first, one cathartic, then another, until at last calomel was given in half tea-spoonful doses, followed by two and sometimes three large spoonsful of castor oil, to produce a single evacuation of the bowels.

Council was now called, and being a physician of the same school, he fully approved of the treatment which the child had received. When interrogated as to the probable result of the case, he replied, "the child cannot recover unless new insides are put into it."

Calomel was left to be given by the nurse *ad libitum*, *ad infinitum*, as affording the only possible chance for saving the child's life, which was despaired of alike by physician and friends. At the end of five days, there being no improvement, the medicine was discontinued and I was sent for. Found the child fearfully emaciated, weighing, at seven months and a half, only six pounds, three pounds less than at birth. On a close examination, no organic lesion was detected. Salivary and urinary secretions active, tongue moist and clean, appetite pretty good, stomach digests well, unless too large an amount of food is given at any given time, when emesis occurs, manifest some degree of intelligence. I was led to regard the case as one of simple constipation, as the result of excessive absorption of intestinal liquids, and a want of normal activity in the secretions of the mucus follicles of the bowels. It was immediately put on the following; Ext. sarsa. ℈jss, aqua ferventis ℥ii, syr. of ipeca ʒii, hydr. bi-chlor. grs. iii, simple syr. ℥ii.

The sarsa. to be dissolved in the water, then add the bi-chloride, being careful that all is dissolved, then add syrups; of this a tea-spoonful to be taken three times daily. The bowels to be kept open with castor oil, until the mixture has time to act. The oil was discontinued at the expiration of two weeks, the mixture alone being sufficient to keep the bowels open. At the end of two weeks more, the dose was reduced one half, in one week more one-fourth the original dose was given, and at the end of two weeks more was discontinued entirely, the discharges being rather too frequent. Meanwhile the child improved rapidly in flesh and appearance. It is now fourteen months old, and in perfect health, weighing eighteen pounds. There has been no recurrence of constipation, defecation occurring with perfect regularity.

The points of interest in this case are: the length of time the constipation continued.

2dly. The point of emaciation to which the child was reduced, being apparently incompatible with life; and

3dly. The efficiency of the remedy in promptly and effectually removing the constipation, and restoring the child to perfect and permanent health. What the precise *modus operandi* of this mixture is, I will not say, though I believe it acts mainly by virtue of its antiseptic and alterative properties. Certain it is that in some way it excites the secretions, restores the equilibrium between absorption and secretion necessary to the maintainance of health. The bi-chloride, I think, is entitled to a higher rank among therapeutical agents than it at present occupies.

I was led to employ it in this case, from having somewhere seen a report of the successful treatment of a similar case by the use of bi-chloride. The manner in which it was used in that case I have forgotten. I believe it was claimed in that report, that the constipation so often attending pregnant females was, in many instances, owing to the same pathological condition, requiring a similar treatment. In this opinion I fully concur.

